# Photoreceptor Mediated Plant Growth Responses: Implications for Photoreceptor Engineering toward Improved Performance in Crops

**DOI:** 10.3389/fpls.2017.01181

**Published:** 2017-07-11

**Authors:** Ophilia I. L. Mawphlang, Eros V. Kharshiing

**Affiliations:** Department of Botany, St. Edmund’s College, Shillong Shillong, India

**Keywords:** plant growth and development, light signaling, plant photoreceptors, photoreceptor engineering, crop productivity

## Abstract

Rising temperatures during growing seasons coupled with altered precipitation rates presents a challenging task of improving crop productivity for overcoming such altered weather patterns and cater to a growing population. Light is a critical environmental factor that exerts a powerful influence on plant growth and development ranging from seed germination to flowering and fruiting. Higher plants utilize a suite of complex photoreceptor proteins to perceive surrounding red/far-red (phytochromes), blue/UV-A (cryptochromes, phototropins, ZTL/FKF1/LKP2), and UV-B light (UVR8). While genomic studies have also shown that light induces extensive reprogramming of gene expression patterns in plants, molecular genetic studies have shown that manipulation of one or more photoreceptors can result in modification of agronomically beneficial traits. Such information can assist researchers to engineer photoreceptors via genome editing technologies to alter expression or even sensitivity thresholds of native photoreceptors for targeting aspects of plant growth that can confer superior agronomic value to the engineered crops. Here we summarize the agronomically important plant growth processes influenced by photoreceptors in crop species, alongwith the functional interactions between different photoreceptors and phytohormones in regulating these responses. We also discuss the potential utility of synthetic biology approaches in photobiology for improving agronomically beneficial traits of crop plants by engineering designer photoreceptors.

## Introduction

“Light exerts a powerful influence on most vegetable tissues, and there can be no doubt that it generally tends to check their growth” – Charles Darwin, 1880 (The Power of Movement in Plants)

Light is a critical environmental factor that influences growth and development in plants. After seed germination in the soil, etiolated growth enables the germinated seedling to grow toward the soil surface in search of light. Upon exposure to light, the seedling undergoes photomorphogenesis characterized by de-etiolation, chlorophyll synthesis and development of chloroplast all of which enable the seedling to establish itself as an independent autotroph. Quality as well as quantity of incident light influence the developmental and growth of plants (Kami et al., 2010; Li J. et al., 2012). Research in plant photobiology has led to the discovery of several light-absorbing photoreceptor proteins that initiate plant responses to light. These include the red/far-red absorbing phytochromes (Chen and Chory, 2011), blue/UV-A absorbing cryptochromes and phototropins (Christie et al., 2015) and UV-B absorbing UVR8 (Rizzini et al., 2011). Most of these photoreceptors except for UVR8 contain more than one member, with each individual member being encoded by a different gene and sharing a high degree of similarity among the individual members of the same family. Higher plants contain multiple phytochromes (phyA to phy E), three cryptochromes (cry1, cry2, and cry3), two phototropins (phot1 and phot2), and one UVR8 photoreceptor. Land plants also contain a family of blue-light absorbing proteins referred to as ZTL/FKF1/LKP2 (ZEITLUPE/FLAVIN-BINDING KELCH REPEAT F-BOX 1/LOV KELCH PROTEIN 2) having a combination of photoreceptor and F-box protein activities within the same protein (Ito et al., 2012). Photoreceptors are widely distributed among land plants, with most of the commonly cultivated crop species reported to contain members of at least one major photoreceptor family (Kharshiing and Sinha, 2015). While most of our understanding on the roles of photoreceptors in plant development has been derived from research on Arabidopsis, there is an increasing interest in photoreceptor-mediated developmental responses in an agricultural environment (Ballaré et al., 1992; Sawers et al., 2005; Hudson, 2008; Wargent and Jordan, 2013; González et al., 2015) since several plant developmental responses involve action of one or more of these photoreceptors.

Plant growth and development involves complex signaling networks which are tightly regulated by genetic and environmental factors. These factors influence a plant’s ability to germinate, adapt, survive, and reproduce in natural conditions. In agriculture, the genetic constitution of different crop species have been altered via various crop breeding programs for enhancing their survival and productivity under different environmental conditions (Osakabe et al., 2011; Slade and Moehs, 2011; Suprasanna and Nakagawa, 2011; Ukai and Nakagawa, 2011). Regulating plant developmental processes such as biomass production, flowering, fruiting and senescence are of particular interest to agricultural scientists for enhancing productivity. Additionally, processes that regulate disease and/or pest resistance (Jeong et al., 2010), starch metabolism (Zeeman et al., 2010), fruit size and quality (Causse et al., 2006), shelf-life of fruits and tubers (Dahmani-Mardas et al., 2010) and plant productivity under high planting index (Warnasooriya and Brutnell, 2014) can also influence productivity of agricultural crops. Various reports have shown that plant photoreceptor action can influence plant growth at different developmental stages including but not limited to seed germination, plant architecture, flowering, reproduction, biomass accumulation and senescence. Plant photoreceptors and/or their signaling components are therefore attractive targets for altering productivity and yield in crop plants for future food and non-food applications (Kharshiing and Sinha, 2015). Here we discuss various photoreceptor-controlled developmental processes that can affect plant productivity and the practical implications for engineering such photoreceptors toward enhancing productivity of crop plants.

## Photoreceptors and Plant Productivity

### Phytochromes

**FIGURE 1:**
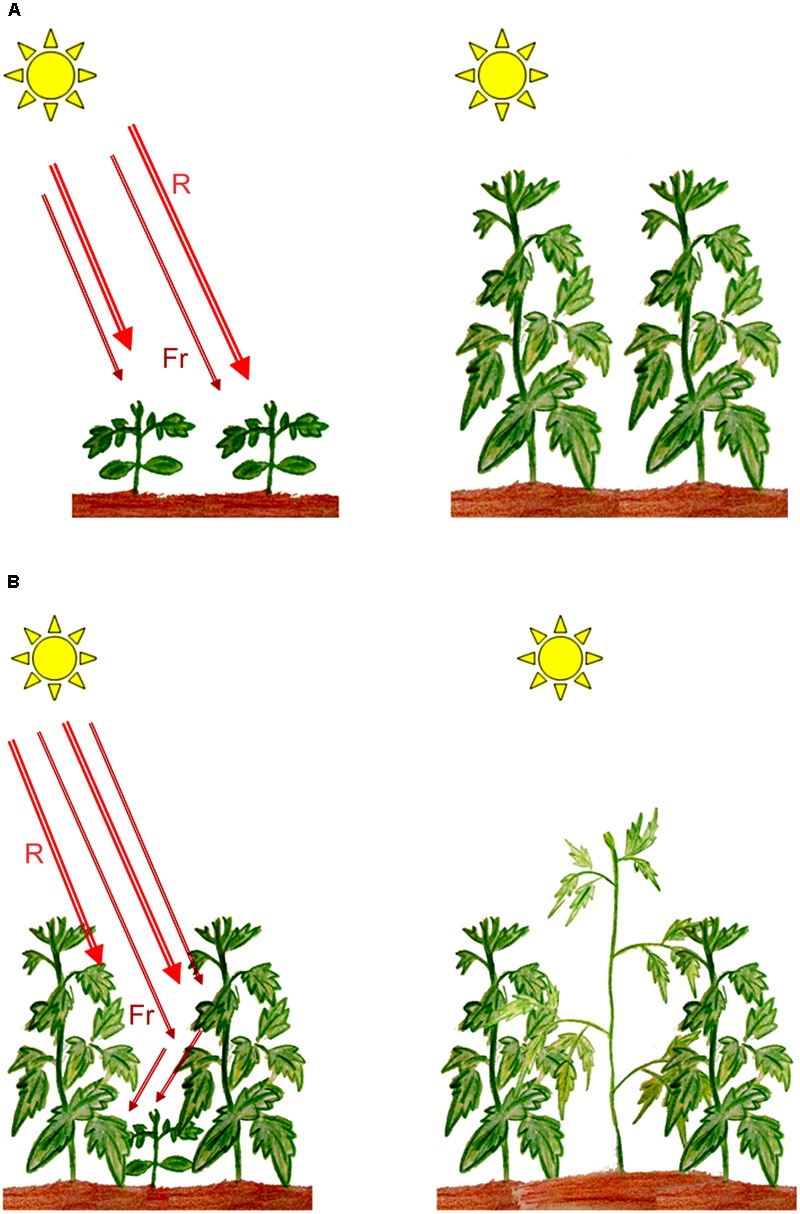
Induction of shade avoidance responses (SAR) in plants growing under shade. **(A)** Plants growing in full sunlight; **(B)** Low Red/Far-red ratios perceived by plants growing under shade resulting in SAR. R, red light; Fr, far-red light. (Artwork by Eros Kharshiing).

Molecular genetics approaches in plant photobiology have led to the isolation and characterization of photoreceptor genes from various species, most of which play critical roles in determining plant survival and development. Phytochromes, which are principal receptors for light in the red/far-red region of the spectrum (600–750 nm), play an essential role in regulating seed germination and seedling establishment in agriculturally important crops such as rice and tomato (Chung and Paek, 2003; Appenroth et al., 2006; Eckstein et al., 2016), while in maize, phytochromes have been implicated to contribute to the transcription of genes essential for photosynthesis (Markelz et al., 2003). Under natural or field conditions, establishment of the emergent seedling post germination is a vital developmental process that determines the ability of the plant to survive, grow, and reproduce. In photosynthesising organisms such as green plants, light is a crucial factor in determining the establishment of an emergent seedling. Maximizing photosynthesis during early development and following the formation of gaps during growth under dense canopies could therefore be critical for seedling survival. Under high plant densities, shade provided by neighboring vegetation triggers a series of developmental changes in growing plants involving several of the known photoreceptors (Ballaré and Pierik, 2017). The actions of phytochromes enables a plant to quantify shade around a seedling’s environment by detecting changes in the Red/Far-red ratios (R:FR) and trigger a series of developmental responses that is thought to provide the plant with a competitive advantage over its neighbors (Schmitt et al., 2003, **Figure 1**). Such responses include stimulation of elongation growth, coupled with reduced leaf development, increased apical dominance, and a reduction in branching (Franklin, 2008). While such developmental plasticity to diminished light, termed as shade avoidance responses (SAR), might increase survival percentage under limiting light conditions, it could result in compromised productivity of crop plants especially in modern intensive cropping methods with high planting densities (Weijschedé et al., 2006; Weinig et al., 2006).

In modern agricultural practices, enhanced yield in food crops such as maize have been achieved by use of varieties that perform optimally at high planting densities but require higher inputs of fertilizer (Warnasooriya and Brutnell, 2014). Modulating the responses of crop plants to vegetative shade for increasing harvestable biomass under high-density planting by manipulating light signaling networks that are fundamental to shade response therefore presents itself as an attractive alternative. Mutant lines harboring lesions in *PHYA* and *PHYB*, two of the more functionally important phytochrome genes in plants, show extreme SAR (Kendrick et al., 1997; Weller et al., 2001; Franklin, 2008; Casal, 2012). The overexpression of these genes could, however, compensate for reduced available light in densely grown crops resulting in increased yield (Robson et al., 1996; Boccalandro et al., 2003; Garg et al., 2006). In Arabidopsis, Devlin et al. (2003) have identified a number of shade responsive genes that are also regulated by phyA and phyB. Recently in Arabidopsis and tomato, phytochromes have been suggested to influence plant biomass along with carbon assimilation and starch metabolism (Kharshiing and Sinha, 2016; Yang et al., 2016). Altering responses of crop plants to light spectral quality by targetting genes such as phytochromes or their signaling components can therefore influence both resource partitioning and growth patterns in crop plants, which would presumably result in higher productivity even under intensive cropping patterns (Warnasooriya and Brutnell, 2014).

### Cryptochromes and Phototropins

Apart from phytochromes, two additional classes of photoreceptors have been reported in plants, which absorb blue/UV-A light, viz., cryptochromes and phototropins (Yu et al., 2010; Christie et al., 2015). In plants, the cryptochrome family photoreceptors participate in several plant process, ranging from photomorphogenesis to entrainment of the circadian clock (Lin, 2002; Botto et al., 2003; Millar, 2003; Giliberto et al., 2005). Developmental processes such as plant height and flowering time which are agronomically important traits are also linked to cryptochrome activity (Mockler et al., 2003; Yu and Lin, 2005; Sharma P. et al., 2014). Since the isolation of the first *CRY* gene from Arabidopsis, *CRYs* have been reported from most major crops investigated (Kharshiing and Sinha, 2015) having roles in seed germination, leaf senescence, stress responses and regulation of transcription (Lopez et al., 2012; Meng et al., 2013; Barrero et al., 2014; Facella et al., 2017). As Arabidopsis is limited as a model plant for examining the molecular networks influencing agronomically important traits (Kimura and Sinha, 2008) several workers have evaluated the role of photoreceptors in other crop models. In *Brassica napus*, an oilseed crop, the overexpression of *CRY1* produced short-statured plants, which confer resistance to wind and water lodging (Sharma P. et al., 2014), providing such plants with an adaptive advantage in these conditions. Tomato plants overexpressing *CRY2* also displayed short hypocotyl and internode length alongwith delayed flowering (Giliberto et al., 2005). Interestingly, in Arabidopsis, cryptochromes have also been reported to promote growth in a shaded environment (Pedmale et al., 2016), suggesting new a molecular target for altering shade responses in plants. Tomato transgenic lines overexpressing *CRY2* overproduced anthocyanins and chlorophyll in leaves alongwith enhanced flavonoids and lycopene content in fruits. In soybean, transgenic lines with upregulated *CRY2a* expression show delayed senescence while senescence is accelerated in lines with downregulated *CRY2a* expression (Meng et al., 2013). Manipulation of *CRY2* also profoundly affects the molecular pathways related to biotic/abiotic stress, photorespiration, photosynthesis, as well as secondary metabolism pathways, such as biosynthesis of phenylpropanoids, phenolic, and flavonoid/anthocyanin in tomato (Lopez et al., 2012). Tomato lines overexpressing *CRY2* show major changes in the rhythmic oscillations of several genes involved in the entrainment of the endogenous clock (Facella et al., 2008) suggesting a role for the photoreceptors in the input to the tomato biological clock. Interestingly, analyses of circadian clock mutants in Arabidopsis, revealed that genes involved in carbohydrate metabolism including starch synthesis and degradation were affected in all mutant lines tested (Graf et al., 2017). This suggests that inputs to the endogenous clock is vital to the proper regulation of critical physiological and developmental responses in plants. Since cryptochromes play critical roles in the entrainment of the endogenous clock in response to light (Millar, 2003), its genetic manipulation could affect agronomically important traits including starch metabolism which would consequently impact productivity in crop plants.

Phototropins are the principal photoreceptors for blue-light phototropism in plants (Briggs and Christie, 2002; Christie et al., 2015). Besides phototropism, phototropins mediate other critical adaptive responses of plants to the surrounding light environment, which serve to enhance the photosynthetic status of the plants. The opening of stomatal pores which allow for exchange of water and carbon dioxide is redundantly controlled by phototropins (Boccalandro et al., 2012; Sharma S. et al., 2014). The movement of chloroplasts in response to different light intensities is also regulated by the phototropins. When plants are exposed to low/weak light conditions, both phot1 and phot2 induce accumulation of the chloroplasts to the upper surface of the palisade mesophyll cells of leaves to maximize photosynthetic light capture. Under strong light conditions, phot2 mediates the rearrangement of chloroplasts parallel to the direction of the light source so as to minimize photo-damage (Briggs and Christie, 2002; Kasahara et al., 2004). In field conditions, Arabidopsis mutants lacking phototropins have been shown to have reduced photosynthesis (Boccalandro et al., 2012), which could be partially due to the inability of the plants to maximally utilize photosynthetically active radiation (PAR) as the lack of phototropin-mediated adjustment of chloroplast position would inhibit optimal capture of PAR. It is also interesting to note that under laboratory conditions, phototropins promote growth in response to blue light under low light environments (Takemiya et al., 2005). While both phot1 and phot2 are involved in growth enhancement, phot1 is more sensitive than phot2 in promoting growth under low blue light, which can affect plant development in natural conditions of low light. On the other hand, phototropin mutants in *Chlamydomonas* have been shown to display reduced fitness under excessive light indicating a role for phototropins in photosynthetic regulation under high light conditions (Petroutsos et al., 2016). While similar findings have not yet been reported for higher plants, such reports underline the versatile roles that photoreceptors play in growth and development of plants.

### UVR8 and ZTL/FKF1/LKP2

Ultraviolet-B radiation (UV-B) is a key component of the radiation environment that is utilized by plants as a signal for UV acclimation and survival in sunlight (Jansen, 2002; Jenkins, 2014). The discovery of the UV-B responsive UVR8 locus in plants (Kliebenstein et al., 2002; Rizzini et al., 2011) and resultant works (reviewed in Tilbrook et al., 2013; Jenkins, 2014) has tremendously progressed our understanding of how this photoreceptor responds to UV-B at the molecular and biochemical levels. While our knowledge of UVR8 function *in vivo* is still at a very nascent stage, its manipulation might have potential applications in crop improvement. The vegetative phase of plant growth is characterized by key developmental processes such as cell division and elongation, directional growth and branching. Low fluence UV-B regulates several of these developmental responses in plants right from seedling to adult stages (Suesslin and Frohnmeyer, 2003; Shinkle et al., 2004; Wargent et al., 2009). In adult plants, morphogenic responses to UV-B include alterations in leaf characteristics such as leaf area, leaf thickness, leaf mass, and stomatal index (Wargent et al., 2009; Robson et al., 2015), Plants exposed to UV-B radiation also show decrease in chlorophyll content and in chla/b ratio (Lidon and Ramalho, 2011) alongwith decrease in photosynthetic efficiency (Lidon et al., 2012). Conversely, promotion of photosynthetic efficiency of Arabidopsis seedlings exposed to elevated levels of UV-B occurs via UVR8 (Davey et al., 2012). Furthermore, lettuce seedlings exposed to UV-B radiation at early stages of development also resulted in increase in productivity and biomass (Tsormpatsidis et al., 2010; Wargent et al., 2011), which could be due to the photoprotective effects of early UV-B exposure. UV-B radiation has also been reported to induce accumulation of secondary metabolites such as flavonoids (Treutter, 2005), which is reportedly controlled by UVR8 (Jenkins, 2014).

Land plants also contain a family of LOV (Light Oxygen or Voltage) photoreceptors referred to as ZEITLUPE/FLAVIN-BINDING KELCH REPEAT, F-BOX1/LOV KELCH PROTEIN2 (ZTL/FKF1/LKP2) having both photoreceptor and F-box protein activities within the same protein (Ito et al., 2012). Interestingly genes coding for this family of photoreceptors are phyllogenetically divided into two groups in dicots and monocots (Boxall et al., 2005; Taylor et al., 2010), suggesting different functions for these genes. However, the high levels of structural conservedness of ZTL and FKF1 homologs among different monocots and dicots may be suggestive of a certain level of functional conservedness of these genes across species (Taylor et al., 2010). Indeed, mutant analyses in Arabidopsis and other species have revealed that ZTL/FKF1/LPK2 regulate similar developmental pathways across different species (Somers et al., 2004; Yon et al., 2016). In Arabidopsis ZTL mutants, presence of FKF1 and LKP2 compensates for the absence of ZTL in as far as in determining circadian rhythm (Baudry et al., 2010) suggesting some level of functional redundancy between these photoreceptors even though they are reported to have distinct roles in photoperiodic flowering (Song et al., 2014). In crop plants, such as soybean *Gm*ZTL3 a homolog of Arabidopsis ZTL has also been suggested to function as a photoreceptor controlling timing of flowering (Xue et al., 2012), a critical developmental response for crop plants. For a comprehensive review on structure and functions of ZTL/FKF1/LKP2 proteins, readers can refer the works of Suetsugu and Wada (2013) and Zoltowski and Imaizumi (2014). **Table 1** provides a summary of the effects of photoreceptors on agronomic traits in crops.

**Table 1:** Agronomic traits in few crops affected by mutations in photoreceptor genes or by altered expression of photoreceptor genes.

Crop	Locus affected		Traits	Reference
*Oryza sativa*	*PHYB*	Single base insertion	Increased drought tolerance, alleviation of chilling induced photoinhibition	Liu et al., 2012; Yang et al., 2013
	*PHYA* *PHYB* *PHYC*	Insertion of retrotransposon *Tos17* Single base insertion Insertion of retrotransposon *Tos17*	Age related resistance to blast fungus	Xie et al., 2011
	*PHYA*	Overexpression of *At PHYA*	Reduced plant height and increased grain yield	Garg et al., 2006
	*CRY2*	Silencing of *CRY2*	Delayed flowering under long-day and short-day	Hirose et al., 2006
*Zea mays*	*PHYB2*	Deletion	Acceleration of flowering under long-day and short-day	Sheehan et al., 2007
*Triticum* sp.	*PHYC*	Single base substitution; nonsense mutation	Acceleration of flowering under long-day	Chen et al., 2014
*Hordeum vulgare*	*CRY1a/b*	Downregulated gene expression	Increased germination percentages	Barrero et al., 2014
*Solanum tuberosum*	*PHYA*	Increased gene expression	Increased tuber formation	Yanovsky et al., 2003
	*PHYB*	Enhanced gene expression	Increased tuber yield at high planting densities	Boccalandro et al., 2003
*Solanum lycopersicum*	*CRY2*	Increased gene expression	Enhanced pigmentation and lycopene content of fruits	Giliberto et al., 2005
	*PHYA* *PHYB1* *PHYB2*	Single base transition Nonsense mutation Three base substitutions, nonsense mutation	Accelerated transition of fruit ripening stages	Gupta et al., 2014
*Glycine max*	*PHYA3*	40 bp deletion	Early flowering and pod maturation	Watanabe et al., 2009
	*CRY1a*	Enhanced gene expression	Early flowering	Zhang et al., 2008
*Brassica* sp.	*CRY1*	Increased gene expression	Reduced plant stature	Sharma P. et al., 2014
*Pisum sativum*	*PHYA*	Single base substitution	Early photoperiod-independent flowering	Weller et al., 2004

## Inter and Intraclass Interactions of Photoreceptors Affecting Plant Development

Since natural light is composed of different wavelengths, higher plant growing under natural conditions will invariably have activation of more than one photoreceptor at the same time. Such simultaneous activation could thus result in signaling networks where action of one photoreceptor can be affected by the activity of other photoreceptors. While some developmental responses in plants are specifically triggered by activation of a single photoreceptor, there are several instances where signaling networks downstream of light perception of more than one photoreceptor of the same or different family are co-ordinately activated to ensure proper growth and development. Such developmental processes include seedling germination (Bertram et al., 2004; Dechaine et al., 2009), photomorphogenesis (Neff and Chory, 1998; Weller et al., 2001; Kami et al., 2010), plant and leaf architecture (Takemiya et al., 2005; Kozuka et al., 2013), flowering (Más et al., 2000; Weller et al., 2001; Mockler et al., 2003; Endo et al., 2016), and fruit quality (Gupta et al., 2014; González et al., 2015) all of which eventually affect plant productivity. The availability of photomorphogenic mutant and transgenic lines in Arabidopsis, rice, tomato, and other model species have provided valuable information into the functional redundancy and interactions of the signaling pathways of different photoreceptors during plant development (**Table 2**). These interactions become especially relevant in the field where plants are subject to uncontrolled environmental conditions of light, temperature, moisture, etc. A detailed understanding of such functional interactions between photoreceptors would further enable researchers to utilize the information for enhancing productivity of crop plants for food and non-food applications.

**Table 2:** Examples of plant developmental processes involving functional interaction of two or more photoreceptors.

Plant function/response	Interacting photoreceptors	Reference
Seed germination	phyA, phyB2 phyA, phyB	Bertram et al., 2004; Lee and Lopez-Molina, 2012
Photomorphogenesis	phyB, cry2 phyA, phyB phyA,phyB,cry1	Más et al., 2000; Weller et al., 2001; Neff and Chory, 1998
Plant architecture	phot1, phot2	Takemiya et al., 2005
Leaf architecture	phyB, phot1, phot2	Kozuka et al., 2013
Stomatal development	phyA, phyB, cry1, cry2	Kang et al., 2009
Flowering/Timing of flowering	phyB, cry2 phyA, phyB phyA, cry1, cry2	Más et al., 2000; Weller et al., 2001; Mockler et al., 2003
Fruiting/Fruit quality	phyA, phyB1, phyB2 phys, crys	Gupta et al., 2014; González et al., 2015

## Photoreceptors and Phytohormones: Light Mediated Hormonal Regulation of Plant Growth Responses

### Seed Germination

Light is an important signal that functions as a developmental switch in germination and photomorphogenesis. Seedling emergence from soil and subsequent photomorphogenic development involve a vast array of photoreceptors which enable the establishment of emergent seedlings. Activation of these photoreceptors by light results in a range of signaling events which co-ordinate plant growth and development that direct seedling emergence from soil and establish them as autotrophs. There is growing evidence that most of these signaling cascades involve interaction of light and phytohormones signaling pathways (Wang et al., 2013). Since the initial proposal of the Cholodny–Went theory regarding the asymmetric distribution of auxin during shoot phototropism (Koepfli et al., 1938), much information has emerged on the links between light perception and hormonal regulation in plant growth responses. In plants seed germination is inhibited by abscisic acid (ABA) while gibberellic acid (GA) induces germination (Jacobsen et al., 2002; Seo et al., 2006). It is now known that light induces seed germination via the interaction of phytochromes and its partner PIF1 (PHYTOCHROME INTERACTING FACTOR 1) also known as PIF3-LIKE 5 (PIL5), which in turn regulates both ABA and GA signaling through the same downstream regulators (Bae and Choi, 2008; Seo et al., 2008; Oh et al., 2009; de Wit et al., 2016). It seems that phytochrome-mediated degradation of PIF1 is therefore a central mechanism by which light induces seed germination by altering ABA and GA metabolism in seeds. PIFs are also implicated to play a role during seedling transition from a skotomorphogenic to a photomorphogenic mode of development in light by regulating GA levels (Oh et al., 2007). In Arabidopsis, many regulators of hormone signaling including auxin and cytokinin (Cluis et al., 2004), ABA (Chen et al., 2008), ethylene (Yu et al., 2013), jasmonic acid (JA) (Prasad et al., 2012), GA (Alabadí et al., 2008), and brassinosteroid (Li and He, 2016) are reported to share a common signaling node involving the basic leucine zipper (bZIP) transcription factor ELONGATED HYPOCOTYL 5 (HY5), whose cellular accumulation involves light signaling transduction through phytochromes and cryptochromes (Osterlund et al., 2000; Wang et al., 2001).

### Flowering Time

Timing of flowering is an essential developmental process in flowering plants that is important for reproduction. In plants, the timing of flowering is determined by the length of the daylight period during a day (or photoperiod). Broadly, flowering plants are categorized into those that flower when length of daylight exceeds a particular length (called critical length) or long-day plants, those that flower when length of daylight is below the critical length or short-day plants and those that flower independent of the critical length or day-neutral plants. phyA, phyB, and cry2 were the first photoreceptors reported to be involved in regulating the timing of flowering in Arabidopsis as well as in crop plants. In rice, mutations in *PHYB* or *PHYC* cause moderate alteration in flowering while lines carrying mutations in *PHYA* coupled with mutations in either *PHYB* or *PHYC* show very early flowering (Takano et al., 2005). Under long-day conditions, both *PHYC* and *PHYB*-null mutants of wheat also exhibit severe delay in flowering (Chen et al., 2014; Pearce et al., 2016). A study of the transcriptomes of *PHYB*-null and *PHYC*-null mutants indicate that phyB also plays a prominent role in regulating GA, BR, auxin, ABA, and ethylene biosynthesis (Pearce et al., 2016), all of which have been implicated in the photoperiodic control of flowering (Galvão and Schmid, 2014). Of these, the regulation of endogenous GA levels may be important for light-induced flowering. Downregulation of the GA biosynthesis gene *GA20OX* showed delayed flowering in FR enriched environment suggesting a role for this gene in phytochrome-dependent flowering (Hisamatsu and King, 2008). While the association of light and auxin signaling is well established, the tomato *pct1-2* mutant having enhanced polar transport of auxin shows delayed phototropism (Kharshiing et al., 2010a,b) as well as increased number of flowers which bloom at the same time as compared to wild type which have lesser flowers and which bloom sequentially (Al-Hammadi et al., 2003). However, the flowers of the *pct1-2* mutant are male sterile because the anthers lack dehiscence. Similarly in Arabidopsis anther dehiscence is regulated by auxin synthesis (Cecchetti et al., 2008) and also involves the auxin transporters ABCB1 and ABCB19 (Cecchetti et al., 2015). The involvement of ABCB19 as a substrate target for phot1 during shoot phototropism in Arabidopsis (Christie et al., 2011) is further illustration of the overlap of photoreceptor and hormone signaling events in plants. Furthermore, while phytochromes are reported to regulate fruit development and ripening in tomato (Gupta et al., 2014), the overproduction of nitric oxide, a secondary messenger in signaling pathways for several plant hormones, in the *shr* mutant of tomato suppresses fruit growth and ripening (Negi et al., 2010; Bodanapu et al., 2016) implying a possible cross-talk of the two signaling pathways.

### Plant Defense Response

**FIGURE 2:**
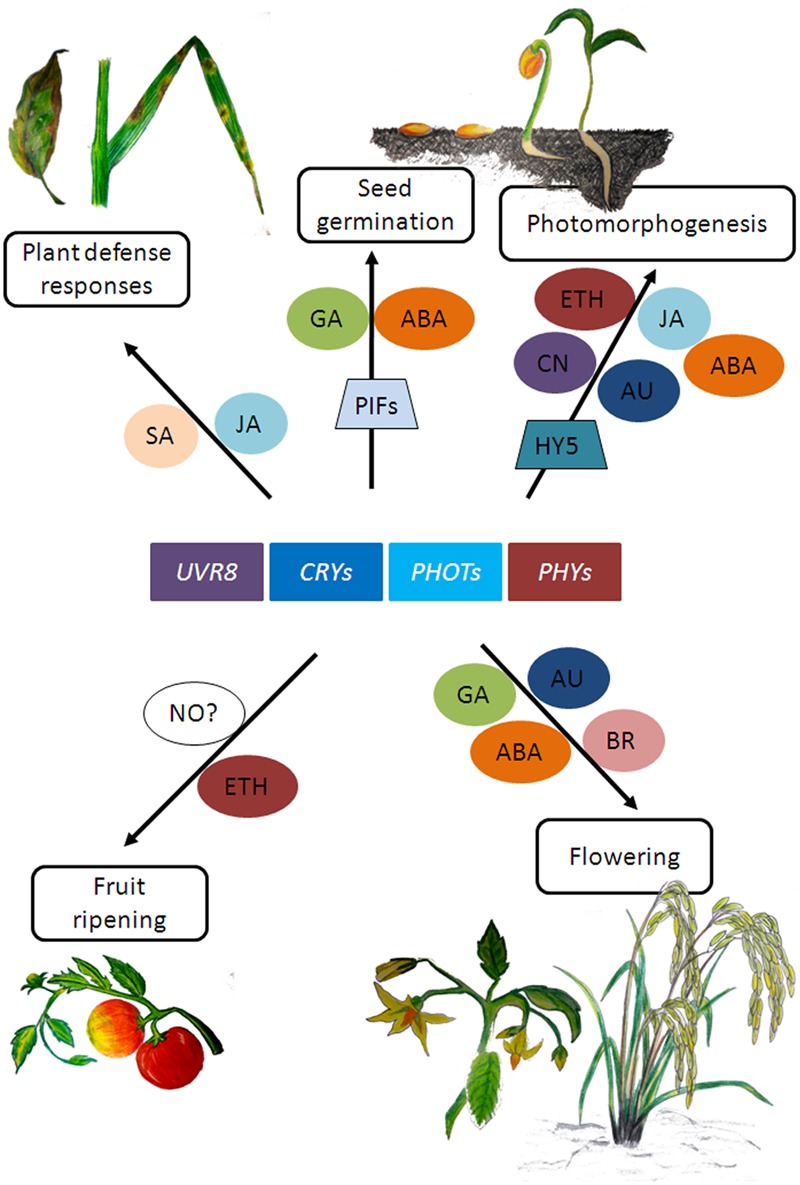
Schematic representation of few plant growth responses involving photoreceptor and hormone signaling. SA, salicylic acid; JA, jasmonic acid; GA, gibberellic acid; ABA, abscisic acid; CN, cytokinins; ETH, ethylene; NO, nitric oxide; BR, brassinosteroids; AU, auxin. (Artwork by Rimeia C. Lyngdoh and Eros Kharshiing).

In plants, while the effect of light on photomorphogenesis and photosynthesis has been known for a long time, it is now becoming increasingly evident that light signaling is also an integral component in determining the outcome of plant-pathogen interactions (Griebel and Zeier, 2008; Ballaré et al., 2012; Demkura and Ballaré, 2012; Erb et al., 2012). Several studies have shown that phytochromes and UVR8 influence plant defense responses (reviewed in Ballaré, 2014; Mazza and Ballaré, 2015; Gommers et al., 2017) while cryptochrome 2 and phototropin 2 have been reported to mediate resistance protein-mediated plant defense against viral but not bacterial pathogens (Jeong et al., 2010). These photoreceptors have been shown to influence plant defense responses by regulating hormone signaling pathways such as that of salicylic acid (SA) and JA (Xie et al., 2011; Moreno and Ballaré, 2014). For a more detailed review on light and hormone signaling, readers can refer to Ballaré (2014), Lucas and Prat (2014); de Wit et al. (2016) and references mentioned therein. The reports mentioned here are few examples of how light and hormone metabolism seem to affect related aspects of plant development that could eventually impact productivity (**Figure 2**). As the mechanisms of signal integration of light and hormone signaling pathways are starting to become clearer, we will be in a better position to understand how light signaling interacts with hormone signaling to regulate traits of agronomic interest in plants.

## Photoreceptor Engineering for Crop Improvement

### Targeted Mutagenesis for Improving Plant Traits

The myriad developmental processes regulated by light suggest that the manipulation of genes involved in light signaling pathways could be a viable tool for crop improvement (Kharshiing and Sinha, 2015). Rapid advancement in genomics is generating new tools for editing genomes which can be utilized for modification of molecular components regulating development in plants (Belhaj et al., 2013; Song et al., 2016). Functional genomics studies, large-scale sequencing and eco-tilling strategies are rapidly identifying polymorphisms between cultivars and landraces resulting in large datasets of molecular diversity among crop plants. Such data is likely to result in the identification of natural alleles of photomorphogenic genes, as well as those that have arisen as a consequence of crop breeding. Genome projects of crops such as rice, maize, tomato, soybean, and others are increasingly generating information which can be used to effect targeted dwarfing, alter SAR, enhance yield and regulate fruiting and ripening in fruit crops by modifying photoreceptors and/or their signaling pathways. Unlike genetically modified transgenics, the upregulation or downregulation of genes in an organism does not necessarily require the introduction of foreign genes into the organism. Several reports on mutation studies also provide evidence that traits in an organism can be modified without the introduction of foreign genes into the system. Many agronomically valuable phenotypes and naturally occurring variants of crop plants have been caused by point, or only a few, mutations. During the last 70 years, more than 3200 crop varieties derived directly as mutant or their progenies have been released worldwide (Pathirana, 2011; Manova and Gruszka, 2015). Many of these mutant-derived varieties have significant economic value such as shorter growth cycle (Ahloowalia et al., 2004), semi-dwarf habit, high harvest index and drought tolerance (D’Souza et al., 2009) and disease resistance (D’Souza et al., 2009; Pathirana, 2011).

Gene targeting via site-directed mutagenesis is a technique that is commonly used in molecular biology to introduce mutations in defined site(s) on the genome. Apart from its utility in studying gene function, the versatility of these techniques also enables researchers to produce gene knockouts or point mutations in plants which can be used for molecular breeding purposes. For a more detailed summary of the various gene targeting strategies in higher plants via site-directed mutagenesis, the reader can refer to Osakabe and Osakabe (2014). While gene targeting in plant photobiology has been commonly utilized for elucidating photoreceptor function and/or signaling, such strategies have been commonly employed for improving the efficiency of reporter genes such as GFP (Cinelli et al., 2000) or iLOV (Christie et al., 2012) which is a derivative of the Light Oxygen Voltage (LOV) domains of phototropin blue-light receptors (Chapman et al., 2008). Similarly, the availability of sequence information of various plant photoreceptors coupled with information from functional genomic studies presents an ideal situation for researchers to engineer photoreceptors or even their downstream signaling partners for improving agronomically valuable traits in crop plants. These could include altering the sensitivity thresholds, photocycles or kinase activity of the different photoreceptor proteins. The emergence of large-scale screening technologies such as TILLING (McCallum et al., 2000; Henikoff et al., 2004; Kurowska et al., 2011) would also allow researchers to couple random mutagenesis with targeted-selected mutagenesis for selecting mutations in photoreceptor genes for subsequent evaluation for desirable traits without the involvement of transgenic modifications.

### Synthetic Biology Approaches to Crop Improvement

For many centuries, humans have been constantly modifying plants that are beneficial to them. With advancements in crop improvement practices, modification of plant characteristics that were beneficial especially in terms of yield, were favorably targeted. For many decades, traditional breeding practices along with mutation breeding programs have been instrumental in modifying agricultural traits in crop plants. However, a growing global population coupled with altered weather patterns, present multifaceted challenges to future agricultural production both for food and non-food applications. Synthetic biology has emerged as a viable technology for rapid, precise, and robust engineering of organisms for useful societal purposes (Shih et al., 2016). Synthetic biology attempts to create user-designed biological systems including plants which can display various characteristics such as responses to nutrition status, infections or to changes in environment. One defining emphasis of synthetic biology is the designed control of gene expression. Through various approaches, gene expression can be controlled at the DNA, RNA, or protein level, depending on the strategy or application. The increasing availability of resources to efficiently inactivate or replace genes in complex organisms such as plants, has led to a revolution in genome editing in the plant science community. Gene editing tools allow targeting of specific DNA sequences within the plant genome thereby enabling researchers to engineer genes and genomes with higher precision than would have been possible earlier. The various tools and technologies utilized in plant synthetic biology have been discussed in previous reviews (Bortesi and Fischer, 2015; Schaeffer and Nakata, 2015; Puchta, 2016; Weeks et al., 2016). While most of the focus of synthetic biology has been on microbes, plant synthetic biology has made rapid progress in recent years. Within the last few years, synthetic biology approaches for regulating plant responses has been successfully demonstrated in several crop species (**Table 3**). As gene editing techniques become more precise, the modulation of photoreceptor activity by engineering designer photoreceptors provides novel opportunities for improving productivity of crop plants. While different engineered photoreceptor systems may require different designs for optimal function (Schmidt and Cho, 2015), such photoreceptor systems can be utilized to determine how the light input affects agronomically beneficial phenotypes.

**Table 3:** Examples of agronomically beneficial traits in some crop plants through application of genome editing technologies.

Synthetic tools	Crop	Trait affected	Reference
Meganuclease	Cotton	Herbicide tolerance	D’Halluin et al., 2013
	Maize	Production of male sterile plants	Djukanovic et al., 2013
TALENs	Potato	Improvement of cold storage and accumulation of reducing sugars in tubers	Clasen et al., 2016
	Soybean	Improved oil quality	Haun et al., 2014
	Rice	Disease resistance	Li T. et al., 2012
	Wheat	Heritable resistance to powdery mildew	Wang et al., 2014
CRISPR/CAS9	Tomato	Early flowering and early ripening	Soyk et al., 2017
	Tomato	Broad-spectrum disease resistance	de Toledo Thomazella et al., 2016
Zinc finger nucleases (ZFN)	Zea mays	Tolerance to multiple herbicides	Shukla et al., 2009
	Zea mays	Resistance to multiple herbicides	Ainley et al., 2013

## Conclusion

Genomic studies have shown that light induces extensive reprogramming of gene expression patterns in plants (Li L. et al., 2012; Petrillo et al., 2014; Perrella and Kaiserli, 2016). The emergence of more precise and robust gene modification technologies provides researchers with exciting options to engineer photoreceptors and/or their signaling components for modulating the response of plants to light inputs. Such targeted gene editing technologies would allow for subsequent engineering of light responses for development of useful agronomic traits in both food and non-food crops. As seen in **Table 1**, alterations in gene function arising due to mutations or altered expression levels of photoreceptors can produce agronomically desirable traits in crops. While research on crops is gaining momentum, the rapid pace of research in Arabidopsis and other established model species is continually contributing large amounts of information on photoreceptor signaling which is yet to be translated to research in crops. However, the information gained from such studies can assist researchers to engineer photoreceptors via genome editing technologies to alter expression or even sensitivity thresholds of native photoreceptors for targeting aspects of plant development that can confer superior agronomic value to the engineered crops. While genome editing in crop plants may have perceived safety concerns (Cardi, 2016) which would influence the future integration of these crops into society (Araki and Ishii, 2015), the myriad of agronomically desirable traits that are developmentally regulated by different photoreceptors either alone or in concert with other photoreceptors and signaling pathways favorably places these genes as most suitable candidates for molecular breeding approaches for enhancing the agronomic value of domesticated lines.

## Author Contributions

EK designed and directed the study, OM and EK carried out the study, EK wrote the manuscript. All authors read and approved the manuscript.

## Conflict of Interest Statement

The authors declare that the research was conducted in the absence of any commercial or financial relationships that could be construed as a potential conflict of interest.
